# Biologic use and treatment patterns in patients with chronic rhinosinusitis with nasal polyps: a US real-world study

**DOI:** 10.1186/s13223-023-00855-7

**Published:** 2023-12-08

**Authors:** Jared Silver, Elizabeth Packnett, Julie Park, Arijita Deb

**Affiliations:** 1 GSK, Durham, NC USA; 2 Merative, Cambridge, MA USA; 3 GSK, Upper Providence, PA USA

**Keywords:** Chronic rhinosinusitis, Corticosteroid use, FESS, Immunotherapy, Sinus surgery, Therapeutics

## Abstract

**Background:**

Several biologics are now approved in the US as add-on treatments for chronic rhinosinusitus with nasal polyps (CRSwNP). This cross-sectional, retrospective, real-world study aimed to characterize treatment patterns and identify predictors of biologic use among patients with CRSwNP.

**Methods:**

Adults in the Merative MarketScan Commercial and Medicare Supplemental Databases with medical claims for CRSwNP were identified June 2018–June 2019 (identification period [IP]). Patient characteristics were collated in the IP and treatment pattern data during the IP plus the following year (July 2019–June 2020; observation period [OP]). Data were stratified by sinus surgery and biologic use.

**Results:**

Of the 5997 eligible patients identified (58% male, mean age 48.1 years), 10.7% (n = 642) used biologics during the OP. More biologic users had common respiratory conditions than non-users, particularly asthma (89.1% vs 35.0%; P < 0.001). Biologic users had fewer diagnostic services but more drug-related services than non-users. Only 11.6% of patients who had sinus surgery used biologics, with most (56.1%) having their first biologic dose before sinus surgery and 12.5% ≤ 30 days after. Oral corticosteroid (OCS) use was higher in biologic users than non-users (all patients: 68.8% vs 42.5%; P < 0.001) and in those with/without sinus surgery. Comorbidities, prior OCS/doxycycline use, and age (< 65 years) increased the odds of biologic use, with asthma increasing the odds 5.46 times (P < 0.001).

**Conclusions:**

Biologic use was more common before first/next sinus surgery and in patients with high unmet need, elucidating predictors of biologic use that could be used in clinical practice.

**Supplementary Information:**

The online version contains supplementary material available at 10.1186/s13223-023-00855-7.

## Background

Chronic rhinosinusitis (CRS), an inflammatory disease of the sinuses, is estimated to affect 2–14% of the US population, with approximately 25–30% of all CRS cases associated with the presence of nasal polyps (CRSwNP) [1–5]. Nasal polyps are inflammatory outgrowths on the lining of nasal passages and sinuses found most frequently associated with CRS [2, 6]. Symptoms of CRSwNP including nasal congestion, rhinorrhea, hyposmia, and facial pain/pressure [7–9] have a substantial negative impact on patients’ health-related quality of life [10].

Management of CRSwNP aims to treat the underlying inflammation and symptoms to improve the patient’s quality of life [8, 9]. Typical first-line standard of care (SoC) treatments include topical intranasal corticosteroids and nasal saline irrigation, as well as antibiotics to address certain types of acute bacterial exacerbations [9]. Oral corticosteroids (OCS) are a short-term option for treating severe symptoms which persist in patients already receiving first-line options, but their long-term benefit is limited due to serious adverse effects [9, 11–13]. If these methods fail to adequately control CRSwNP, patients may undergo endoscopic sinus surgery to remove NP, which has been shown to significantly improve symptoms [8, 9]. However, recurrence of NP is common following sinus surgery, with studies showing 40% of patients experiencing recurrence 18 months after surgery and 37% having multiple surgeries over a 12-year period [14, 15].

Several biologics have been approved for severe asthma and since 2018 [16–21]; a number of these agents are now indicated for treatment of CRSwNP: dupilumab, omalizumab, and mepolizumab [22–25]. With the recent approvals of biologics for CRSwNP, precise guidelines on how to use these treatments to achieve optimal patient outcomes are evolving. Recent International Consensus Statement on Allergy and Rhinology (ICAR) 2021 guidelines include recommendations for use of specific biologics in severe CRSwNP [9].

Understanding real-world treatment patterns is particularly important as new therapies are approved to assess how these therapies fit into existing treatment paradigms. However, to our knowledge, there is currently no real-world evidence on patterns of biologic use in patients with CRSwNP or which patients are most likely to have them prescribed. This real-world study uses one of the largest US proprietary claims databases (MarketScan) to assess treatment patterns during a period when biologics were first approved for CRSwNP and already in use for patients with asthma and comorbid CRSwNP. Using these data, the study aimed to provide an understanding of patterns and predictors of biologic use among patients with CRSwNP in relation to other SoC treatment lines in the context of current treatment recommendations.

## Methods

### Study design and patient eligibility

This was a cross-sectional, retrospective, real-world cohort study (GSK ID: 214150) using the Merative MarketScan Commercial Database and Medicare Supplemental Database (study period: June 30, 2018, to June 30, 2020). The MarketScan and Medicare Databases include medical records of cost, use, and outcomes data for healthcare services performed in both inpatient and outpatient settings.

Patients were identified based on their earliest non-diagnostic medical claim for CRSwNP (index date) between June 30, 2018, and June 01, 2019 (identification period). Eligible patients had ≥ 2 non-diagnostic medical claims for CRSwNP ≥ 1 day apart during the identification period, were ≥ 18 years of age on the date of the first non-diagnostic CRSwNP medical claim, and were continuously enrolled during the study period. Non-diagnostic medical claims excluded claims with procedure codes for lab tests or radiologic procedures (e.g., magnetic resonance imaging, X-ray, or ultrasound) used to diagnose or rule out a condition. CRSwNP medical claims were inferred by NP diagnosis codes (e.g., ICD-10-CM J33xx).

**Fig. 1:**
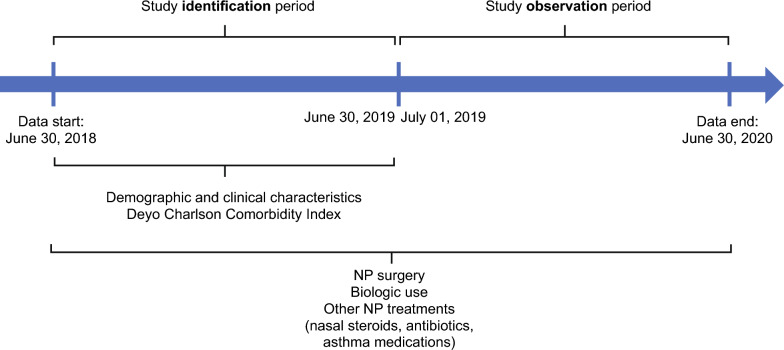
Study design. All study variables (including those needed for patient selection) were collected from the databases using enrollment records, and ICD-10-CM, ICS-10-PCS, 4th edition CPT, Healthcare Common Procedure Coding System, and National Drug Codes, as appropriate. Eligible patients were identified as having sinus surgery if they had a CPT or ICD-10-PCS code for sinus surgery on a medical claim during the observation period. The duration between biologic use and surgery was assessed before and after surgery. The study period was limited based on the data available at the time of the study. CRSwNP, chronic rhinosinusitis with nasal polyps

Baseline demographics and clinical characteristics of identified patients were collected during the identification (June 30, 2018 to June 30, 2019) period while data on treatment patterns were collected over the entire study period and assessed during the observation period, between July 1, 2019, and June 30, 2020 (Fig. 1).

All database records were de-identified and fully compliant with US patient confidentiality requirements, including the Health Insurance Portability and Accountability Act (HIPAA) of 1996. The databases were evaluated and certified by an independent third party to follow the HIPAA statistical de-identification standard.

### Outcomes

Study outcomes included baseline demographics, clinical characteristics, and treatment patterns, all of which were stratified by sinus surgery (sinus surgery and no sinus surgery) and biologic use (biologic use and no biologic use). Clinical characteristics included Deyo-Charlson Comorbidity Index (DCI), clinical conditions, asthma exacerbations, and CRSwNP-related healthcare resource utilization (HCRU). Asthma exacerbations were identified if patients had either an outpatient claim with a diagnosis of asthma and ≥ 1 prescription of systemic corticosteroids ± 5 days after the asthma claim, or if patients had an inpatient hospital claim with a primary diagnosis of asthma. An exacerbation recorded within 14 days of a previous exacerbation was counted with the previous exacerbation as a single episode.

Treatment pattern assessments included biologic use and non-biologic use, and the temporal relationship between surgery and biologic use. Patients were identified as receiving sinus surgery based on CPT or ICD-10-PCS procedure codes (Additional file 1). Evidence of biologic use (benralizumab, dupilumab, mepolizumab, omalizumab, and reslizumab), OCS use for any reason, CRSwNP-related OCS use (prednisone, betamethasone, cortisone, dexamethasone, hydrocortisone, methylprednisolone, prednisolone, triamcinolone, budesonide, deflazacort, paramethasone, and fludrocortisone), and other CRSwNP-related pharmacologic treatment (intranasal corticosteroids and oral antibiotic use) were identified in patients with ≥ 1 pharmacy or medical claim using National Drug Codes (NDC) or Healthcare Common Procedure Coding System (HCPCS) codes. Biologic treatment duration was calculated as the total number of days of supply or clinical benefit for any biologic used. CRSwNP-related OCS use was identified in patients with OCS use in proximity to a CRSwNP-related inpatient claim (± 5 days), a CRSwNP-related outpatient claim without an asthma claim (± 5 days), or a sinus surgery claim (± 30 days). The proximity of biologic use to sinus surgery was evaluated by calculating the proportion of patients with earliest biologic use before or on/after their earliest sinus surgery. Among these patients, the proportion with first biologic use within 30 days before or on/after sinus surgery was analyzed, respectively.

A logistic regression model was used to identify independent predictive factors for biologic use during the observation period in patients without biologic use during the identification period. Biologic use during the identification period and observation period were highly associated, so only patients without biologic use during the identification period were included in the model. Covariates included demographics (age group < 65 or ≥ 65 years, sex), baseline clinical characteristics (allergic rhinitis, asthma, atopic dermatitis, chronic rhinosinusitus, and gastroesophageal reflux disease [GERD]), and prior treatment/diagnostics (doxycycline use, endoscopy procedure, sinus surgery, OCS use, and sinus computed tomography [CT] scan).

### Statistical analysis

Descriptive analyses were used to describe demographics, clinical characteristics, HCRU, and biologic and CRSwNP treatment use. Post hoc analyses were conducted to describe CRSwNP-related OCS use and presence and frequency of asthma exacerbations in the identification and observation periods. Chi-squared tests were used for categorical variables and t-tests for continuous variables, and P < 0.05 was considered statistically significant. Odds ratios (ORs) and 95% confidence intervals (CIs) were calculated to describe the relationship between biologic use and the independent variables included in the logistic regression models during the observation period.

## Results

### Patient population

**Fig. 2:**
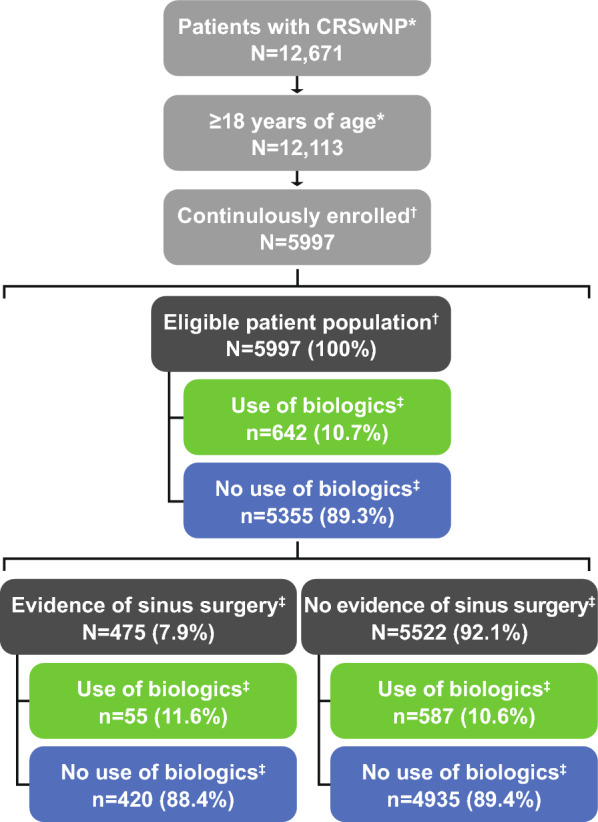
Patient sample selection. Patients in the Merative MarketScan Commercial or Medicare Supplemental Databases with ≥ 2 non-ruleout (i.e., non-diagnostic) medical claims with a diagnosis of CRSwNP (as inferred by NP diagnosis codes; e.g., ICD-10-CM J33xx) in any position ≥ 1 day apart between 6/30/2018 and 6/30/2019. Reasons for biologic use were not available in the claims database; therefore, biologics may have been prescribed for conditions other than CRSwNP. *During identification period (6/30/2018–6/30/2019); ^†^during the study period (6/30/2018–6/30/2020); ^‡^during the observation period (7/1/2019–6/30/2020). CRSwNP, chronic rhinosinusitis with nasal polyps

Of the 12,671 patients identified with CRSwNP, 5997 met the study eligibility criteria (Fig. 2). There were 642 (10.7%) biologic users, 5355 (89.3%) non-biologic users, 475 (7.9%) who had sinus surgery, and 5522 (92.1%) with no evidence of sinus surgery.

### Patient demographics and clinical characteristics

Among the total population, the majority were male (57.9%) and had a mean (standard deviation [SD]) age of 48.1 (13.1) years (Table 1). There was a significant difference in the distribution of patients by age group between biologic users and non-users (P = 0.002). There were more biologic users than non-users in the middle-aged groups (35–44, 45–54, and 55–64 years) for the total population and those with no sinus surgery. More biologic users than non-biologic users lived in urban areas (92.1% vs 88.8%; P = 0.021). The proportion of biologic users covered by commercial insurance was 96.3%, with 11.0% of patients with commercial insurance receiving biologics; 3.7% of patients had Medicare supplemental coverage with 6.5% of patients with Medicare supplemental coverage receiving biologics (Table 1).

**Table 1:** Patient demographics stratified by sinus surgery and biologic use

Demographics	All patients	All patients			Sinus surgery			No sinus surgery		
	N = 5997	Biologic use	No biologic use	P-value	Biologic use^†^	No biologic use	P-value	Biologic use	No biologic use	P-value
		N = 642	N = 5355		N = 55	N = 420		N = 587	N = 4935	
**Age, years, mean (SD)**	48.1 (13.1)	48.8 (11.4)	48.0 (13.3)	0.171	45.4 (11.4)	45.7 (13.9)	0.886	49.1 (11.4)	48.2 (13.2)	0.129
**Age group, years, n (%)**										
18–34	977 (16.3)	78 (12.1)	899 (16.8)	**0.002**	12 (21.8)	96 (22.9)	0.741	66 (11.2)	803 (16.3)	**0.002**
35–44	1227 (20.5)	148 (23.1)	1079 (20.1)		14 (25.5)	80 (19.0)		134 (22.8)	999 (20.2)	
45–54	1697 (28.3)	192 (29.9)	1505 (28.1)		15 (27.3)	118 (28.1)		177 (30.2)	1387 (28.1)	
55–64	1731 (28.9)	201 (31.3)	1530 (28.6)		13 (23.6)	101 (24.0)		188 (32.0)	1429 (29)	
65–74	229 (3.8)	15 (2.3)	214 (4.0)		1 (1.8)	17 (4.0)		14 (2.4)	197 (4.0)	
≥ 75	136 (2.3)	8 (1.2)	128 (2.4)		0 (0)	8 (1.9)		8 (1.4)	120 (2.4)	
**Male, n (%)**	3472 (57.9)	335 (52.2)	3137 (58.6)	**0.002**	27 (49.1)	255 (60.7)	0.099	308 (52.5)	2882 (58.4)	**0.006**
**Geographic region, n (%)**										
Northeast	1043 (17.4)	104 (16.2)	939 (17.5)	0.253	6 (10.9)	67 (16.0)	0.501	98 (16.7)	872 (17.7)	0.296
North Central	1426 (23.8)	164 (25.5)	1262 (23.6)		10 (18.2)	99 (23.6)		154 (26.2)	1163 (23.6)	
South	2685 (44.8)	278 (43.3)	2407 (44.9)		28 (50.9)	194 (46.2)		250 (42.6)	2213 (44.8)	
West	821 (13.7)	96 (15.0)	725 (13.5)		11 (20.0)	56 (13.3)		85 (14.5)	669 (13.6)	
Unknown	22 (0.4)	0 (0)	22 (0.4)		0 (0)	4 (1.0)		0 (0)	18 (0.4)	
**Residence, n (%)**										
Urban	5347 (89.2)	591 (92.1)	4756 (88.8)	**0.021**	51 (92.7)	365 (86.9)	0.617	540 (92.0)	4391 (89.0)	**0.044**
Rural	628 (10.5)	51 (7.9)	577 (10.8)		4 (7.3)	51 (12.1)		47 (8.0)	526 (10.7)	
Unknown	22 (0.4)	0 (0)	22 (0.4)		0 (0)	4 (1.0)		0 (0)	18 (0.4)	
**Insurance plan type, n (%)**										
Comprehensive/indemnity	251 (4.2)	30 (4.7)	221 (4.1)	0.943	0 (0)	16 (3.8)	0.629	30 (5.1)	205 (4.2)	0.891
EPO/PPO	2964 (49.4)	317 (49.4)	2647 (49.4)		26 (47.3)	209 (49.8)		291 (49.6)	2438 (49.4)	
POS/POS with capitation	392 (6.5)	42 (6.5)	350 (6.5)		4 (7.3)	25 (6.0)		38 (6.5)	325 (6.6)	
HMO	851 (14.2)	96 (15.0)	755 (14.1)		10 (18.2)	55 (13.1)		86 (14.7)	700 (14.2)	
CDHP/HDHP	1431 (23.9)	147 (22.9)	1284 (24.0)		15 (27.3)	109 (26.0)		132 (22.5)	1175 (23.8)	
Other/unknown	108 (1.8)	10 (1.6)	98 (1.8)		0 (0)	6 (1.4)		10 (1.7)	92 (1.9)	
**Payer, n (%)**										
Commercial	5628 (93.8)	618 (96.3)	5010 (93.6)	**0.007**	54 (98.2)	395 (94.0)	0.342	564 (96.1)	4615 (93.5)	**0.015**
Medicare supplemental	369 (6.2)	24 (3.7)	345 (6.4)		1 (1.8)	25 (6.0)		23 (3.9)	320 (6.5)	

Demographics and medical insurance were measured on the earliest CRSwNP diagnosis date during the patient identification period (6/30/2018–6/30/2019). Patients were stratified by biologic and non-biologic use during the observation period (7/1/2019–6/30/2020). P-values in bold signify P < 0.05 for comparison within individual categories for biologic versus non-biologic use in each cohort (i.e., all patients, sinus surgery, no sinus surgery)

CDHP, consumer-driven health plan; CRSwNP, chronic rhinosinusitis with nasal polyps; EPO, exclusive provider organization; HDHP, high deductible health plan; HMO, health maintenance organization; POS, point of service; PPO, preferred provider organization; SD standard deviation

**Fig. 3:**
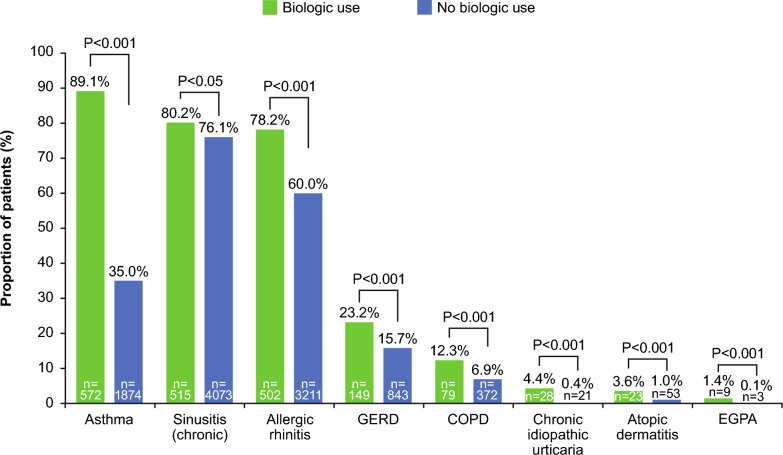
Proportion of patients with comorbid conditions for biologic and non-biologic users. Clinical characteristics were measured on the earliest CRSwNP diagnosis date during the study identification period. In addition to the comorbid conditions shown, significantly more biologic users had respiratory infections than non-biologic users. COPD, chronic obstructive pulmonary disease; CRSwNP, chronic rhinosinusitis with nasal polyps; EGPA, eosinophilic granulomatosis with polyangiitis; GERD, gastroesophageal reflux disease

In the total population, biologic users had a significantly higher mean DCI score than non-biologic users (1.2 vs 0.7, P < 0.001; Table 2). Also, 62.1% of patients had  ≥ 3 comorbid conditions, with this proportion significantly higher for biologic users compared with non-biologic users (84.7% vs 59.4%; P < 0.001). This was independent of whether patients had previous sinus surgery (83.6% vs 66.7%; P < 0.023) or not (84.8% vs 58.8%; P < 0.001). In total, 46.0% of the total population had ≥ 3 common respiratory conditions (including acute sinusitis, allergic rhinitis, asthma, chronic rhinosinusitus, and respiratory infections), and this proportion was significantly higher for biologic users compared with non-biologic users (73.7% vs 42.7%; P < 0.001), whether patients had sinus surgery (78.2% vs 49.8%; P = 0.001) or not (73.3% vs 42.1%; P < 0.001). Furthermore, a higher proportion of biologic users compared with non-biologic users had ≥ 1 medical claim for asthma (89.1% vs 35.0%; P < 0.001) and had experienced ≥ 1 (24.8% vs 18.4%; P = 0.001) and ≥ 2 asthma exacerbations (26.8% vs 16.8%; P = 0.012). Among all patients, the three most common comorbidities were chronic rhinosinusitus (76.5%), allergic rhinitis (61.9%), and asthma (40.8%). The proportion of patients with these respiratory comorbidities, along with GERD and COPD, was significantly higher for patients with biologic use compared with non-biologic use (Fig. 3).

**Table 2:** Clinical characteristics and HCRU of patients with CRSwNP, stratified by surgery and biologic use

	All patients	All patients			Sinus surgery			No sinus surgery		
	N = 5997	Biologic use N = 642	No biologic use N = 5355	P-value	N = 55 Biologic use	N = 420 No biologic use	P-value	N = 587 Biologic use	N = 4935 No biologic use	P-value
DCI [44, 45] mean (SD)	0.8 (1.1)	1.2 (1.0)	0.7 (1.1)	** < 0.001**	1 (0.7)	0.8 (1.2)	0.143	1.2 (1.0)	0.7 (1.1)	** < 0.001**
**Clinical conditions, n (%)**										
Asthma	2446 (40.8)	572 (89.1)	1874 (35.0)	** < 0.001**	44 (80.0)	148 (35.2)	** < 0.001**	528 (89.9)	1726 (35.0)	** < 0.001**
Allergic rhinitis	3713 (61.9)	502 (78.2)	3211 (60.0)	** < 0.001**	46 (83.6)	262 (62.4)	**0.002**	456 (77.7)	2949 (59.8)	** < 0.001**
Atopic dermatitis	76 (1.3)	23 (3.6)	53 (1.0)	**< 0.001**	0 (0)	7 (1.7)	1	23 (3.9)	46 (0.9)	** < 0.001**
Chronic idiopathic urticaria	49 (0.8)	28 (4.4)	21 (0.4)	** < 0.001**	3 (5.5)	1 (0.2)	**0.005**	25 (4.3)	20 (0.4)	** < 0.001**
COPD	451 (7.5)	79 (12.3)	372 (6.9)	** < 0.001**	9 (16.4)	36 (8.6)	0.064	70 (11.9)	336 (6.8)	** < 0.001**
Diabetes	573 (9.6)	60 (9.3)	513 (9.6)	0.849	4 (7.3)	44 (10.5)	0.459	56 (9.5)	469 (9.5)	0.977
Eosinophilic esophagitis	26 (0.4)	5 (0.8)	21 (0.4)	0.191	0 (0)	0 (0)	1	5 (0.9)	21 (0.4)	0.189
EGPA	12 (0.2)	9 (1.4)	3 (0.1)	**< 0.001**	0 (0)	0 (0)	1	9 (1.5)	3 (0.1)	** < 0.001**
GERD	992 (16.5)	149 (23.2)	843 (15.7)	**< 0.001**	14 (25.5)	69 (16.4)	0.097	135 (23.0)	774 (15.7)	** < 0.001**
Hypertension	1816 (30.3)	190 (29.6)	1626 (30.4)	0.689	11 (20.0)	121 (28.8)	0.170	179 (30.5)	1505 (30.5)	0.999
Malignant neoplasm of respiratory and intrathoracic organs	25 (0.4)	0 (0)	25 (0.5)	0.103	0 (0)	4 (1.0)	1	0 (0)	21 (0.4)	0.158
Respiratory infections	1566 (26.1)	196 (30.5)	1370 (25.6)	**0.007**	19 (34.5)	129 (30.7)	0.564	177 (30.2)	1241 (25.1)	0.009
Rheumatoid arthritis	55 (0.9)	3 (0.5)	52 (1.0)	0.206	0 (0)	4 (1.0)	1	3 (0.5)	48 (1.0)	0.269
Rhinosinusitis (acute)	2034 (33.9)	233 (36.3)	1801 (33.6)	0.178	24 (43.6)	171 (40.7)	0.679	209 (35.6)	1630 (33.0)	0.211
Rhinosinusitis (chronic)	4588 (76.5)	515 (80.2)	4073 (76.1)	**0.019**	48 (87.3)	359 (85.5)	0.721	467 (79.6)	3714 (75.3)	**0.022**
SLE	13 (0.2)	2 (0.3)	11 (0.2)	**0.642**	0 (0)	0 (0)	1	2 (0.3)	11 (0.2)	0.641
**Number of comorbid conditions (among all listed above), n (%)**										
0	155 (2.6)	1 (0.2)	154 (2.9)	** < 0.001**	0 (0)	2 (0.5)	**0.023**	1 (0.2)	152 (3.1)	** < 0.001**
1	760 (12.7)	18 (2.8)	742 (13.9)		1 (1.8)	57 (13.6)		17 (2.9)	685 (13.9)	
2	1357 (22.6)	79 (12.3)	1278 (23.9)		8 (14.5)	81 (19.3)		71 (12.1)	1197 (24.3)	
≥ 3	3725 (62.1)	544 (84.7)	3181 (59.4)		46 (83.6)	280 (66.7)		498 (84.8)	2901 (58.8)	
Number of common respiratory conditions,* n (%)										
0	289 (4.8)	2 (0.3)	287 (5.4)	** < 0.001**	0 (0)	7 (1.7)	**0.001**	2 (0.3)	280 (5.7)	** < 0.001**
1	1143 (19.1)	31 (4.8)	1112 (20.8)		3 (5.5)	75 (17.9)		28 (4.8)	1037 (21.0)	
2	1804 (30.1)	136 (21.2)	1668 (31.1)		9 (16.4)	129 (30.7)		127 (21.6)	1539 (31.2)	
≥ 3	2761 (46.0)	473 (73.7)	2288 (42.7)		43 (78.2)	209 (49.8)		430 (73.3)	2079 (42.1)	
**Asthma exacerbations, n (%)**^*†*^										
Medical claim for asthma	2446 (40.8)	572 (89.1)	1874 (35.0)	** < 0.001**	44 (80.0)	148 (35.2)	** < 0.001**	528 (89.9)	1726 (35.0)	** < 0.001**
≥ 1 asthma exacerbation	487 (19.9)	142 (24.8)	345 (18.4)	**0.001**	14 (31.8)	33 (22.3)	0.197	128 (24.2)	312 (18.1)	**0.002**
**Number of exacerbations**										
1	391 (80.3)	104 (73.2)	287 (83.2)	**0.048**	8 (57.1)	19 (57.6)	0.915	96 (75.0)	268 (85.9)	**0.012**
2	67 (13.8)	24 (16.9)	43 (12.5)		6 (42.9)	11 (33.3)		18 (14.1)	32 (10.3)	
3	17 (3.5)	7 (4.9)	10 (2.9)		0 (0)	1 (3.0)		7 (5.5)	9 (2.9)	
4	7 (1.4)	4 (2.8)	3 (0.9)		0 (0)	2 (6.1)		4 (3.1)	1 (0.3)	
≥ 5	5 (1.0)	3 (2.1)	2 (0.6)		0 (0)	0 (0)		3 (2.3)	2 (0.6)	
≥ 2 asthma exacerbations	96 (19.7)	38 (26.8)	58 (16.8)	**0.012**	6 (42.9)	14 (42.4)	0.978	32 (25.0)	44 (14.1)	**0.006**
**CRSwNP-related HCRU, n (%)**										
Endoscopy services	4358 (72.7)	388 (60.4)	3970 (74.1)	** < 0.001**	39 (70.9)	314 (74.8)	0.539	349 (59.5)	3656 (74.1)	** < 0.001**
Sinus CT scan services	1337 (22.3)	86 (13.4)	1251 (23.4)	** < 0.001**	14 (25.5)	147 (35.0)	0.16	72 (12.3)	1104 (22.4)	** < 0.001**
Office-administered CRSwNP-related drug services	328 (5.5)	207 (32.2)	121 (2.3)	** < 0.001**	17 (30.9)	10 (2.4)	** < 0.001**	190 (32.4)	111 (2.2)	** < 0.001**
Outpatient pharmacy prescriptions	5373 (89.6)	616 (96.0)	4757 (88.8)	** < 0.001**	53 (96.4)	391 (93.1)	0.561	563 (95.9)	4366 (88.5)	** < 0.001**

Clinical characteristics and healthcare resource utilization were measured on the earliest CRSwNP diagnosis date during the patient identification period (6/30/2018–6/30/2019). Patients were stratified by biologic and non-biologic use during the observation period (7/1/2019–6/30/2020). P-values in bold signify P < 0.05 for comparison within individual categories for biologic versus non-biologic use in each cohort (i.e., all patients, sinus surgery, no sinus surgery)

^*^Common respiratory conditions include chronic rhinosinusitus, allergic rhinitis, asthma, acute sinusitis, and respiratory infections; ^†^asthma exacerbations were identified if either of the following criteria were met: outpatient or emergency department visit with a diagnosis of asthma AND ≤ 1 dispensing of systemic corticosteroids ± 5 days after the encounter, or inpatient hospital admissions with a diagnosis of asthma as a primary diagnosis. An exacerbation recorded within 14 days of a previous exacerbation was counted with the previous exacerbation as part of a single exacerbation episode

COPD, chronic obstructive pulmonary disease; CRSwNP, chronic rhinosinusitis with nasal polyps; CT, computed tomography; DCI, Deyo-Charlson Comorbidity Index; EGPA, eosinophilic granulomatosis with polyangiitis; GERD, gastroesophageal reflux disease; HCRU, healthcare resource utilization; SD, standard deviation; SLE, systemic lupus erythematosus

### CRSwNP-related HCRU

In the total population, most patients (72.7%) had CRSwNP-related endoscopies and sinus CT scans (22.3%). There was a lower rate of endoscopies (60.4%) among biologic users compared with non-biologic users (74.1%; P < 0.001) and sinus CT scans (13.4% vs 23.4%, respectively; P < 0.001; Table 2). A lower rate of endoscopy and sinus CT scans in biologic users versus non-biologic users was also observed among patients both with and without sinus surgery, although the differences were greater in patients without sinus surgery. However, compared with non-users, more biologic users had CRSwNP-related office-administered service use (32.2% vs 2.3%; P < 0.001) and CRSwNP-related outpatient pharmacy prescriptions (96.0% vs 88.8%; P < 0.001).

### Patterns of biologic and CRSwNP-related treatment use

**Fig. 4:**
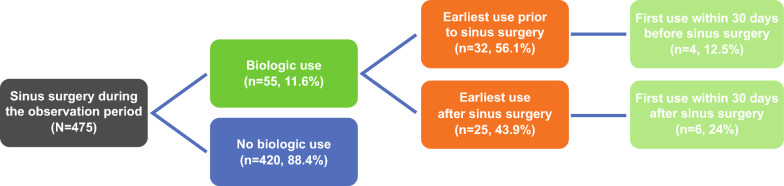
First biologic use before and after surgery. “Patients with biologic use” was measured at any point during the study period among patients with sinus surgery during the observation period. Complete patient history before the study period was not available in the database; therefore, patients recorded as having no biologic use or sinus surgery may have received these before the study period

Among the 475 patients who had sinus surgery during the observation period, 55 (11.6%) had used biologics at some point during the study period (Fig. 4). Of this population, 32 (56.1%) had their earliest biologic use before their earliest sinus surgery, whereas 25 (43.9%) had their earliest biologic use on or after their earliest sinus surgery. Four patients (12.5%) had their earliest biologic use within 30 days before sinus surgery, and six (24.0%) had earliest biologic use within 30 days on or after the earliest sinus surgery. During the observation period, the mean (SD) number of days between first biologic use and earliest sinus surgery was 277.8 (171.6), and between earliest sinus surgery and first biologic use after surgery was 91.4 (64.0). The mean (SD) number of biologic claims was similar between patients with sinus surgery (8.0 [7.0]) and those without surgery (8.6 [5.8]). Biologic users with sinus surgery had fewer mean (SD) days on biologic therapy (152.6 [118.0]) than those without sinus surgery (182.5 [116.2]).

Among the total population, 865 patients (14.4%) had CRSwNP-related OCS use and 1992 (33.2%) used intranasal corticosteroids during the observation period (Table 3). Among the total population, the proportion of patients using OCS was higher in biologic users compared with non-users (68.8% vs 42.5%; P < 0.001). Similarly, use of OCS was also higher among biologic users versus non-users in those with sinus surgery (87.3% vs 72.9%, respectively; P < 0.021) and those without sinus surgery (67.1% vs 39.9%, respectively; P < 0.001). Similar differences were also observed between biologic and non-biologic users for CRSwNP-related OCS use during the observation period, but the difference was not significant in the sinus surgery cohort (67.3% vs 59.0%, respectively; P = 0.242). In the 30 days before earliest sinus surgery, OCS (49.1% vs 30.9%, respectively; P = 0.007) and intranasal corticosteroids (20.0% vs 10.2%, respectively; P = 0.032) were used more frequently in biologic users than non-users. Conversely, in the 30 days after earliest sinus surgery, OCS and intranasal corticosteroid use did not significantly differ between these cohorts.

**Table 3:** Non-biologic treatments for patients with CRSwNP, stratified by surgery and biologic use

		All patients			Sinus surgery			No sinus surgery		
	All patients	Biologic use	No biologic use	P-value	Biologic use	No biologic use	P-value	Biologic use	No biologic use	P-value
	N = 5997	N = 642	N = 5355		N = 55	N = 420		N = 587	N = 4935	
**Patients with corticosteroid use, n (%)**										
All OCS use*	2717 (45.3)	442 (68.8)	2275 (42.5)	** < 0.001**	48 (87.3)	306 (72.9)	**0.021**	394 (67.1)	1969 (39.9)	** < 0.001**
CRSwNP-related OCS*	865 (14.4)	136 (21.2)	729 (13.6)	** < 0.001**	37 (67.3)	248 (59.0)	0.242	99 (16.9)	481 (9.7)	** < 0.001**
Intranasal corticosteroids	1992 (33.2)	282 (43.9)	1710 (31.9)	** < 0.001**	29 (52.7)	185 (44.0)	0.224	253 (43.1)	1525 (30.9)	** < 0.001**
**Patients with antibiotic (oral) use, n (%)**										
Amoxicillin	1836 (30.6)	234 (36.4)	1602 (29.9)	** < 0.001**	28 (50.9)	199 (47.4)	0.622	206 (35.1)	1403 (28.4)	** < 0.001**
Azithromycin	1010 (16.8)	147 (22.9)	863 (16.1)	** < 0.001**	14 (25.5)	74 (17.6)	0.16	133 (22.7)	789 (16.0)	** < 0.001**
Clarithromycin	169 (2.8)	36 (5.6)	133 (2.5)	** < 0.001**	4 (7.3)	27 (6.4)	0.772	32 (5.5)	106 (2.1)	** < 0.001**
Doxycycline	837 (14.0)	122 (19.0)	715 (13.4)	** < 0.001**	14 (25.5)	87 (20.7)	0.419	108 (18.4)	628 (12.7)	** < 0.001**
Erythromycin	4 (0.1)	1 (0.2)	3 (0.1)	0.364	0 (0)	0 (0)	> 0.999	1 (0.2)	3 (0.1)	0.362
**Treatments used in the 30 days before earliest sinus surgery, n (%)**										
OCS	154 (33.0)	–	–	–	27 (49.1)	127 (30.9)	**0.007**	–	–	–
Intranasal corticosteroids	53 (11.4)	–	–	–	11 (20.0)	42 (10.2)	**0.032**	–	–	–
Antibiotics (oral)	78 (16.7)	–	–	–	8 (14.5)	70 (17.0)	0.643	–	–	–
**Treatments used in the 30 days on or after earliest sinus surgery n, (%)**										
OCS	125 (26.8)	–	–	–	15 (27.3)	110 (26.8)	0.936	–	–	–
Intranasal corticosteroids	83 (17.8)	–	–	–	10 (18.2)	73 (17.8)	0.939	–	–	–
Antibiotics (oral)	123 (26.4)	–	–	–	16 (29.1)	107 (26.0)	0.629	–	–	–

Non-biologic treatments during the observation period (7/1/2019–6/30/2020). P-values in bold signify P < 0.05 for comparison within individual categories for biologic versus non-biologic use in each cohort (i.e., all patients, sinus surgery, no sinus surgery). Complete patient history was not available in the database; therefore, patients recorded as having no treatment may have received these before the study period

^*^OCS use based on patients with ≥ 1 pharmacy or medical claim for OCS

CRSwNP, chronic rhinosinusitis with nasal polyps; OCS, oral corticosteroid

### Predictors of biologic use

**Fig. 5:**
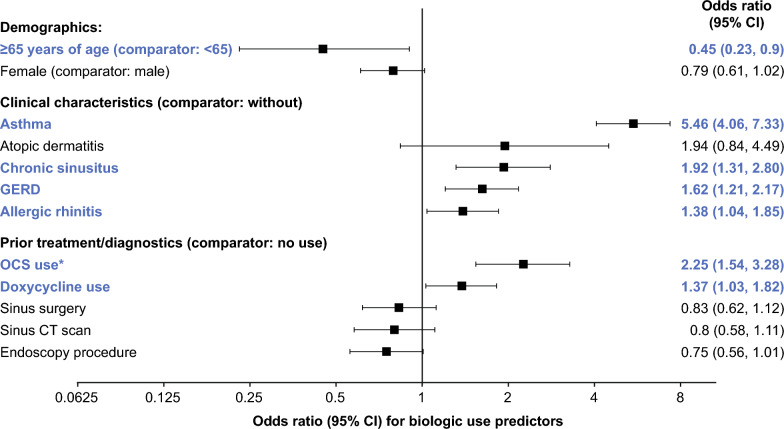
Predictors of biologic use among patients without biologic use during the identification period (N = 5610). All covariates with P < 0.05 for entry into the model using stepwise regression are shown. Covariates and odds ratios in bold and blue text signify P < 0.05 from the regression model. *OCS use based on patients with ≥ 1 pharmacy claim for OCS. CI, confidence interval; CT, computed tomography; GERD, gastroesophageal reflux disease; OCS, oral corticosteroid

Logistic regression analysis of patients without biologic use during the identification period found that the presence versus non-presence of comorbid asthma at baseline increased the odds of using biologic therapy 5.46 times (P < 0.0001; Fig. 5). Other predictive factors associated with significantly higher odds of biologic use included prior OCS use (OR 2.25), chronic rhinosinusitus (OR 1.92), GERD (OR 1.62), prior doxycycline use (OR 1.37), and allergic rhinitis (OR 1.38). Patients ≥ 65 versus < 65 years of age had an OR of 0.45 of using biologics.

## Discussion

This cross-sectional, retrospective, real-world study, conducted in one of the largest claims databases in the US, provides valuable insights into the use of Although asthma severity biologic therapies in patients with CRSwNP during a focused point in time when biologics were first gaining US approval for CRSwNP. Retrospective evaluation of treatment patterns during such a time is particularly useful as this is when new paradigms of care often emerge, allowing assessment of how newly approved biologics begin to fit into everyday clinical practice. Furthermore, these early observations provide the opportunity for the medical community to understand how onsite practice patterns compare with current treatment guidelines.

In line with current treatment guidelines on biologic intervention, this study identified asthma and prior OCS/doxycycline treatment as predictive factors of biologic use. [7, 9, 26]. The other predictive factors identified (allergic rhinitis, chronic rhinosinusitus, GERD, and age < 65 years) extend the profile of patients likely to use biologics. To the best of our knowledge, this is the first study to provide predictors of biologic use in patients with CRSwNP in a quantitative fashion using real-world data from clinical practice. To date, literature that includes patient markers has largely focused on understanding those that predicted response to biologics, rather than biologic use [27–29]. Publications that refer to markers in terms of patient suitability for receiving biologics do so qualitatively [30, 31]. Therefore, this study provides a unique perspective on the clinical use of biologics in CRSwNP, which may help clinicians better understand their patients’ therapeutic needs and the likelihood of them requiring biologics later in their treatment pathway. This could facilitate earlier determination of the appropriate multidisciplinary team input and treatment, monitoring and communication strategy required for patients with high unmet needs.

Patient demographics were similar to other database and clinical trials involving patients with CRSwNP [1, 10, 32–35]. There were a number of key demographic differences between biologic users and non-users. Patients under 65 years of age were over 50% more likely to use biologics than those aged over 65 years, and patients with Medicare Supplemental coverage had approximately 40% lower use of biologics than patients with commercial coverage, which could reflect caution from physicians in prescribing biologics to older patients[36] or differences in insurance coverage of medications in the Medicare Supplemental and Commercial populations. These differences may also explain why biologic users were more likely to live in urban areas and have claims captured in the Commercial database. For example, urban areas in the US frequently contain a greater proportion of people under 65 years of age than rural areas, while the Commercial database includes the under-65 working population versus Medicare, which includes retirees [37, 38]. Potential disparities in access to biologics within urban versus non-urban settings may also be a contributing factor [39].

In this study, biologic users more commonly had comorbid asthma and other clinical conditions than non-biologic users. Indeed, patients with comorbid asthma without biologic use during the study identification period were over fivefold more likely to receive biologics than those without comorbid asthma. This is in accordance with European Position Paper on Rhinosinusitis and Nasal Polyps (EPOS) 2020, and ICAR 2021 criteria for biologic selection, which suggest consideration of the use of biologics in patients with comorbid asthma [7, 9, 26]. In addition, comorbid asthma could possibly be the primary indication for which patients were receiving the biologic, since the biologics included in this study are approved to treat one or more conditions, including severe asthma [16, 18–21]. Although asthma severity was not recorded in this study, the proportion of patients who had ≥ 2 asthma exacerbations provides an estimate of those with severe asthma, as per European Respiratory Society/American Thoracic Society guidelines, which include exacerbation criteria in their definition of severe asthma [40, 41]. As such, only 27% of biologic users had ≥ 2 asthma exacerbations, suggesting most biologic use was not for severe asthma. Beyond asthma, GERD was also identified as a predictor of biologic use, possibly relating to increased likelihood of type 2 inflammation (i.e., eosinophilia and high levels of interleukin-4/5/13) and therefore biologic selection, in patients with CRSwNP and GERD [7, 8, 42].

Biologic users more commonly used OCS and other CRSwNP-related treatments as well as drug-related services than non-biologic users; however, use of diagnostic services (e.g., endoscopy and sinus CT scan services) was lower in biologic users than non-users. OCS response can indicate the presence of type 2 inflammation and thus the likelihood of response to biologics which target the type 2 pathway (i.e., immunoglobulin E, interleukin-4/5/13) [7, 8, 26]. Furthermore, as per treatment paradigm/guidance recommendations, advancing to biologic treatment requires a confirmed diagnosis of uncontrolled severe disease for which a patient has received systemic corticosteroids in the previous 2 years [7, 26]. Greater diagnostic service utilization in non-biologic users versus biologic users could be related to more recent CRSwNP diagnosis requiring more frequent monitoring, such as value judgment [9]. Also, biologic users may have reduced need for diagnostics due to clinical response. Greater use of drug-related services in biologic users compared with non-users is potentially related to associated biologic prescriptions and administrations. Use of OCS and doxycycline, which were predictive factors of biologic use, indicates more severe disease, higher disease burden, and unmet need in biologic users. This aligns with ICAR 2021 guidelines that recommend use of biologics in severe CRSwNP in circumstances when other treatment options have failed, which would include OCS and doxycycline as short-term early interventions before considering biologics [9].

Aside from differences in additional treatments between biologic users and non-users, there were also variations in biologic use between patients with versus without sinus surgery. Although the mean number of biologic claims was similar between these two groups, patients with surgery had fewer days on biologic therapy, suggesting sufficient symptomatic relief and/or enhanced disease control compared with those without surgery. During the observation period less than 10% of all patients received sinus surgery, and only 12% of those that did used biologics. The low frequency of surgery observed may primarily reflect the limited observation period in this study; capturing only recent surgeries represents those with a more current and active burden of disease. This low incidence of surgery combined with low biologic use might suggest that when biologics were first being used for CRSwNP, they were less likely to be used in patients with a disease burden high enough to necessitate surgery, or that “salvage surgery under biological protection” was not routinely considered due to lack of empirical evidence to support this approach [7]. For patients who used biologics after surgery, they did so within a relatively short time (~ 3 months). This potentially reflects a failure of surgery, or early attempts to combine therapies. Therefore, biologic use within 6 months after surgery might mean these patients were having reoccurrence of NP, which is common in CRSwNP [15], or they are simply receiving biologics as an add-on maintenance therapy for inadequately controlled CRSwNP [18–20]. For non-biologic treatments, OCS and intranasal corticosteroid use were higher in biologic users versus non-users in the 30 days before surgery but not in the 30 days after surgery. Preoperative OCS use might have been a more common approach in biologic users versus non-users, possibly due to the higher disease burden that biologic users likely represent [8, 13]. Together, these findings suggest biologics are frequently used alongside OCS but less commonly with sinus surgery. In the cases where biologics and surgery were used together, biologics were used soon after surgical intervention, which could indicate a more aggressive approach to care.

This study had some limitations. Several relate to the use of databases, such as data entry errors and therefore potential underestimates, although provider reimbursement schemes minimize this risk. Similarly, the results are subject to data coding limitations, which might explain why the percentage of patients with chronic rhinosinusitus is low, considering NP without CRS is rare. Categorizing both acute and chronic respiratory conditions together when assessing the proportion of patients with multiple common respiratory conditions may have overestimated the proportion of patients with overlapping conditions, compared with if overlapping chronic inflammatory conditions had been assessed separately. There was an absence of patient data before the identification period, so patients recorded as having ‘no’ or ‘earliest’ biologic use, sinus surgery, or other CRSwNP treatments may have received these before the study period; a longer pre-study observation period would have allowed for the capture of these records. The source population receives private insurance, which may not reflect the general population in the US. There were no data on lifestyle factors or CRSwNP symptoms that might have influenced treatment decisions. Beyond database-related limitations, the exact reasons for biologic use were mostly unknown as several biologics reported in this study were not approved for CRSwNP during the study period. Despite this, biologics were likely prescribed for one of the comorbid conditions and less so for chronic idiopathic urticaria, atopic dermatitis, and Eosinophilic granulomatosis with polyangiitis (EGPA) that had low patient numbers. A similar limitation would apply to the CRSwNP-related OCS use, although we used a robust surrogate assessment to identify CRSwNP-related OCS use (e.g., OCS claims must be within ± 5 days CRSwNP-related inpatient claim) it is possible that some claims may have been inaccurately identified. In addition, the use of over-the-counter treatments, such as intranasal corticosteroids, was likely underestimated [43, 44]. A longer study period would have provided greater insights towards the relationship between biologics and SoC. As this study covered a time period close to the first approval of biologics for the treatment of CRSwNP, market factors, such as payer policies, may have influenced the study findings. However, it is worth noting that more recent data would potentially be subject to confounding from the COVID-19 pandemic; biologic use may have changed since the period described in this study.

## Conclusions

In conclusion, these results suggest that during an early period of biologic introduction for patients with CRSwNP, biologic therapy was prescribed mostly to those with severe disease, as indicated by an increased number of comorbidities and common OCS use compared with non-users. These findings suggest that the early treatment paradigm reserved biologic use for those with the highest unmet need, an approach consistent with current clinical recommendations. A range of predictive factors of biologic use related to patient characteristics were identified These may help clinicians better understand the treatment needs of their patients and facilitate earlier identification of those who may require biologic therapy, ultimately helping to establish a tailored, personalized plan for individual patient monitoring and care among the multidisciplinary team. Combined use of sinus surgery and biologics was uncommon, which may reflect clinicians’ reluctance to use both options, low biologic use in patients with disease burden sufficient to necessitate surgery, or limitations of the dataset/analysis which may not capture, for example, all recent historical surgery. The rationale for the treatment patterns described here is speculative, and future studies are required to have a more complete understanding of long-term treatment patterns and clinical outcomes of biologic use in CRSwNP beyond the early biologic approval period. Furthermore, reassessment of real-world biologic use over time will be important to see how the care model for CRSwNP evolves.

### Supplementary Information

**Additional file 1: ** Supplementary methods.

## Data Availability

GSK makes available anonymized individual participant data and associated documents from interventional clinical studies that evaluate medicines, upon approval of proposals submitted to: https://www.gsk-studyregister.com/en/

## Abbreviations

CRSwNP: Chronic rhinosinusitus with nasal polyps
CRS: Chronic rhinosinusitis
SoC: Standard of care
OCS: Oral corticosteroids
NP: Nasal polyps
ICAR: International Consensus Statement on Allergy and Rhinology
HIPAA: Health Insurance Portability and Accountability Act
DCI: Included Deyo-Charlson Comorbidity Index
HCRU: Healthcare resource utilization
NDC: National Drug Codes
GERD: Gastroesophageal reflux disease
CT: Computerized tomography
CIs: Confidence intervals
EPOS: European Position Paper on Rhinosinusitis and Nasal Polyps
EGPA: Eosinophilic granulomatosis with polyangiitis

## References

[1] Ference EH, Reddy SR, Tieu R, Gokhale S, Park S, LeCocq J (2020). Burden of Nasal Polyps in the United States. OTO Open..

[2] Stevens WW, Schleimer RP, Kern RC (2016). Chronic Rhinosinusitis with Nasal Polyps. J Allergy Clin Immunol Pract.

[3] Bhattacharyya N, Gilani S (2018). Prevalence of potential adult chronic rhinosinusitis symptoms in the United States. Otolaryngol Head Neck Surg.

[4] Soler ZM, Mace JC, Litvack JR, Smith TL (2012). Chronic rhinosinusitis, race, and ethnicity. Am J Rhinol Allergy.

[5] Hirsch AG, Stewart WF, Sundaresan AS, Young AJ, Kennedy TL, Scott Greene J (2017). Nasal and sinus symptoms and chronic rhinosinusitis in a population-based sample. Allergy.

[6] Newton JR, Ah-See KW (2008). A review of nasal polyposis. Ther Clin Risk Manag.

[7] Bachert C, Han JK, Wagenmann M, Hosemann W, Lee SE, Backer V (2021). EUFOREA expert board meeting on uncontrolled severe chronic rhinosinusitis with nasal polyps (CRSwNP) and biologics: Definitions and management. J Allergy Clin Immunol.

[8] Fokkens WJ, Lund VJ, Hopkins C, Hellings PW, Kern R, Reitsma S (2020). European Position Paper on Rhinosinusitis and Nasal Polyps 2020. Rhinology.

[9] Orlandi RR, Kingdom TT, Smith TL, Bleier B, DeConde A, Luong AU (2021). International consensus statement on allergy and rhinology: rhinosinusitis 2021. Int Forum Allergy Rhinol.

[10] Khan A, Vandeplas G, Huynh TMT, Joish VN, Mannent L, Tomassen P (2019). The Global Allergy and Asthma European Network (GALEN rhinosinusitis cohort: a large European cross-sectional study of chronic rhinosinusitis patients with and without nasal polyps. Rhinology.

[11] Sedaghat AR (2017). Chronic rhinosinusitis. Am Fam Physician.

[12] Waljee AK, Rogers MA, Lin P, Singal AG, Stein JD, Marks RM (2017). Short term use of oral corticosteroids and related harms among adults in the United States: population based cohort study. BMJ.

[13] Jaffuel D, Fabry-Vendrand C, Darnal E, Wilczynski O, Pain E, Bourdin A (2021). Perception of oral corticosteroids in adult patients with asthma in France. J Asthma.

[14] DeConde AS, Mace JC, Levy JM, Rudmik L, Alt JA, Smith TL (2017). Prevalence of polyp recurrence after endoscopic sinus surgery for chronic rhinosinusitis with nasal polyposis. Laryngoscope.

[15] Calus L, Van Bruaene N, Bosteels C, Dejonckheere S, Van Zele T, Holtappels G (2019). Twelve-year follow-up study after endoscopic sinus surgery in patients with chronic rhinosinusitis with nasal polyposis. Clin Transl Allergy.

[16] Benralizumab PI. FASENRA (benralizumab) injection, for subcutaneous use. https://www.accessdata.fda.gov/drugsatfda_docs/label/2017/761070s000lbl.pdf. September 2022.

[17] Brusselle GG, Koppelman GH (2022). Biologic Therapies for Severe Asthma. N Engl J Med.

[18] Dupilumab PI. DUPIXENT (dupilumab) injection, for subcutaneous use. https://www.regeneron.com/downloads/dupixent_fpi.pdf. (2022) August 2022.

[19] Mepolizumab PI. NUCALA (mepolizumab) for injection, for subcutaneous use. https://gskpro.com/content/dam/global/hcpportal/en_US/Prescribing_Information/Nucala/pdf/NUCALA-PI-PIL-IFU-COMBINED.PDF. (2021) August 2022.

[20] Omalizumab PI. XOLAIR (omalizumab) injection, for subcutaneous use. https://www.gene.com/download/pdf/xolair_prescribing.pdf. (2021) August 2022.

[21] Reslizumab PI. CINQAIR (reslizumab) injection, for intravenous use. https://www.accessdata.fda.gov/drugsatfda_docs/label/2016/761033lbl.pdf. (2016) September 2022.

[22] GSK. GSK announces FDA approval for Nucala (mepolizumab) for use in adults with chronic rhinosinusitis with nasal polyps. https://www.gsk.com/en-gb/media/press-releases/gsk-announces-fda-approval-for-nucala-mepolizumab-for-use-in-adults-with-chronic-rhinosinusitis-with-nasal-polyps/. (2021) September 2022.

[23] Novartis. Novartis receives EC approval for new Xolair indication to treat severe chronic rhinosinusitis with nasal polyps. https://www.novartis.com/news/media-releases/novartis-receives-ec-approval-new-xolair-indication-treat-severe-chronic-rhinosinusitis-nasal-polyps. (2020) September 2022.

[24] Regeneron. FDA approves dupixent (dupilumab) for chronic rhinosinusitis with nasal polyposis. https://investor.regeneron.com/news-releases/news-release-details/fda-approves-dupixentr-dupilumab-chronic-rhinosinusitis-nasal. (2019) September 2022.

[25] Cai S, Xu S, Lou H, Zhang L (2022). Comparison of different biologics for treating chronic rhinosinusitis with nasal polyps: a network analysis. J Allergy Clin Immunol Pract.

[26] Fokkens WJ, Lund V, Bachert C, Mullol J, Bjermer L, Bousquet J (2019). EUFOREA consensus on biologics for CRSwNP with or without asthma. Allergy.

[27] Meier EC, Schmid-Grendelmeier P, Steiner UC, Soyka MB (2021). Real-life experience of monoclonal antibody treatments in chronic rhinosinusitis with nasal polyposis. Int Arch Allergy Immunol.

[28] Cardell L-O, Stjärne P, Jonstam K, Bachert C (2020). Endotypes of chronic rhinosinusitis: Impact on management. J Allergy Clin Immunol.

[29] Gevaert P, Lang-Loidolt D, Lackner A, Stammberger H, Staudinger H, Van Zele T (2006). Nasal IL-5 levels determine the response to anti-IL-5 treatment in patients with nasal polyps. J Allergy Clin Immunol.

[30] Bachert C, Zhang N, Hellings PW, Bousquet J (2018). Endotype-driven care pathways in patients with chronic rhinosinusitis. J Allergy Clin Immunol.

[31] Platt MP, Brook CD (2021). Choosing the right patient for biologic therapy in chronic rhinosinusitis with nasal polyposis: endotypes, patient characteristics, and defining failures of standard therapy. Otolaryngol Clin North Am.

[32] Han JK, Bachert C, Fokkens W, Desrosiers M, Wagenmann M, Lee SE (2021). Mepolizumab for chronic rhinosinusitis with nasal polyps (SYNAPSE): a randomised, double-blind, placebo-controlled, phase 3 trial. Lancet Respir Med.

[33] Bachert C, Mannent L, Naclerio RM, Mullol J, Ferguson BJ, Gevaert P (2016). Effect of subcutaneous dupilumab on nasal polyp burden in patients with chronic sinusitis and nasal polyposis: a randomized clinical trial. JAMA.

[34] Bachert C, Han JK, Desrosiers M, Hellings PW, Amin N, Lee SE (2019). Efficacy and safety of dupilumab in patients with severe chronic rhinosinusitis with nasal polyps (LIBERTY NP SINUS-24 and LIBERTY NP SINUS-52): results from two multicentre, randomised, double-blind, placebo-controlled, parallel-group phase 3 trials. Lancet.

[35] Bachert C, Han JK, Desrosiers MY, Gevaert P, Heffler E, Hopkins C (2022). Efficacy and safety of benralizumab in chronic rhinosinusitis with nasal polyps: A randomized, placebo-controlled trial. J Allergy Clin Immunol.

[36] Borren NZ, Ananthakrishnan AN (2019). Safety of biologic therapy in older patients with immune-mediated diseases: a systematic review and meta-analysis. Clin Gastroenterol Hepatol.

[37] Watson Health. IBM MarketScan Research Databases for life sciences researchers. https://www.ibm.com/downloads/cas/OWZWJ0QO. September 2022.

[38] Smith AS, Trevelyan E. The Older Population in Rural America: 2012–2016. American Community Survey Reports: U.S. Census Bureau, 2019.

[39] Peterman NJ, Vashi A, Govan D, Bhatia A, Vashi T, Kaptur B (2022). Evaluation of access disparities to biologic disease-modifying antirheumatic drugs in rural and urban communities. Cureus.

[40] Chung KF, Wenzel SE, Brozek JL, Bush A, Castro M, Sterk PJ (2014). International ERS/ATS guidelines on definition, evaluation and treatment of severe asthma. Eur Respir J.

[41] Lommatzsch M, Virchow JC (2014). Severe asthma: definition, diagnosis and treatment. Deutsches Arzteblatt international.

[42] Kia L, Hirano I (2015). Distinguishing GERD from eosinophilic oesophagitis: concepts and controversies. Nat Rev Gastroenterol Hepatol.

[43] Bridgeman MB (2017). Overcoming barriers to intranasal corticosteroid use in patients with uncontrolled allergic rhinitis. Integr Pharm Res Pract.

[44] American Academy of Allergy Asthma & Immunology. Over-The-Counter Allergy Nasal Steroid Sprays - What Does It Mean For Patients? Available from: https://www.aaaai.org/tools-for-the-public/conditions-library/allergies/triamcinolone-nasal-spray#:~:text=The%20U.S.%20Food%20and%20Drug,purchase%20a%20nasal%20steroid%20spray. (2020) September 2020.

[45] Deyo RA, Cherkin DC, Ciol MA (1992). Adapting a clinical comorbidity index for use with ICD-9-CM administrative databases. J Clin Epidemiol.

