# Supplementary material for: Biologic use and treatment patterns in patients with chronic rhinosinusitis with nasal polyps: a US real-world study
Source: Allergy Asthma Clin Immunol. 2023 Dec 8;19:104. doi: 10.1186/s13223-023-00855-7 (PMC10709958; doi:10.1186/s13223-023-00855-7)
Supplement: Supplementary file 1 — Additional file 1: Supplementary methods. [file 13223_2023_855_MOESM1_ESM.docx]

## Supplement

### Supplementary methods

- All database records were de-identified and fully compliant with US patient confidentiality requirements, including the Health Insurance Portability and Accountability Act of 1996. The databases have been evaluated and certified by an independent third party to follow the HIPAA statistical de-identification standard. The databases were certified to satisfy the conditions set forth in Sections 164.514 (a)-(b)1ii of the Health Insurance Portability and Accountability Act privacy rule regarding the determination and documentation of statistically de-identified data.
- All study variables (including those used for patient selection) were collected from the database using enrolment records, International Classification of Diseases, Tenth Revision, Clinical Modification (ICD-10-CM) codes, International Classification of Diseases, Tenth Revision, Procedural Coding System (ICD-10-PCS) codes, Current Procedural Terminology, 4th edition (CPT) codes, HCPCS codes, and NDC, as appropriate.
- Sinus surgery procedure codes included polypectomy, functional endoscopic sinus surgery, radical nasalization with removal of the middle turbinate, balloon sinuplasty, lavage by cannulation, and sinusotomy.
